# Diagnostic delays for breathlessness in primary care: a qualitative study to investigate current care and inform future pathways

**DOI:** 10.3399/BJGP.2022.0475

**Published:** 2023-04-18

**Authors:** Gillian E Doe, Marie T Williams, Stacey Chantrell, Michael C Steiner, Natalie Armstrong, Ann Hutchinson, Rachael A Evans

**Affiliations:** Department of Respiratory Sciences, University of Leicester, Leicester, UK.; University of South Australia, Adelaide, Australia.; Respiratory Theme, National Institute for Health and Care Research (NIHR) Leicester Biomedical Research Centre, University Hospitals of Leicester NHS Trust, Leicester, UK.; Department of Respiratory Sciences, University of Leicester; respiratory consultant physician, Respiratory Theme, NIHR Leicester Biomedical Research Centre, University Hospitals of Leicester NHS Trust, Leicester, UK.; Department of Health Sciences, University of Leicester, Leicester, UK.; Wolfson Palliative Care Research Centre, Hull York Medical School, University of Hull, Hull, UK.; Department of Respiratory Sciences, University of Leicester; respiratory consultant physician, Respiratory Theme, NIHR Leicester Biomedical Research Centre, University Hospitals of Leicester, Leicester, UK.

**Keywords:** breathlessness, diagnosis, primary care, qualitative research

## Abstract

**Background:**

Evidence about the delays to diagnosis for patients presenting with breathlessness is lacking.

**Aim:**

To explore current care of patients with breathlessness through the experiences of adults presenting with chronic breathlessness who are awaiting a diagnosis and the experiences of primary care clinicians.

**Design and setting:**

Qualitative study with adults presenting with chronic breathlessness and clinicians across 10 general practices.

**Method:**

Semi-structured interviews were conducted with patients and clinicians. Participants were recruited from a feasibility cluster randomised controlled trial investigating a structured diagnostic pathway for breathlessness. An interview guide explored experiences of help seeking for breathlessness, the diagnostic process, and associated health care. Transcripts were analysed using thematic analysis supported by NVivo software.

**Results:**

Interviews were conducted with 34 patients (mean age 68 years, standard deviation [SD] 10.8, of whom 20 were female [59%]) and 10 clinicians (mean 17 years of experience, SD 6.3, of whom five were female [50%]). Five themes were identified: recognising and validating symptoms of breathlessness is an important first step; clinical decision making for breathlessness is complex; difficult conversations arise when a disease-related diagnosis is not confirmed; disease management rather than symptom management is prioritised by clinicians; and patient experience is influenced by clinician communication style.

**Conclusion:**

The findings indicate potential explanations for delays to diagnosis for patients with chronic breathlessness. Interventions are needed to enhance symptom recognition, include alternative approaches to incremental investigation, and expand the concept of diagnosis beyond a disease label to improve communication, with the ultimate aim of earlier diagnosis and management to improve patient outcomes.

## INTRODUCTION

Chronic breathlessness is a common, distressing symptom with a prevalence of 9%–11% in the general population,^1^^,^^2^ increasing to 25% in older age.^3^ Chronic breathlessness syndrome is described as breathlessness persisting despite optimal treatment of the underlying cause, and which causes disability.^4^ This complex symptom negatively affects physical function, quality of life, and survival,^5^^,^^6^ frequently leading to primary and emergency care consultations.^7^^,^^8^ Patients presenting with chronic breathlessness may receive a diagnosis for conditions including cardiorespiratory disease, anxiety, depression, and obesity.^9^^,^^10^ Multimorbidity is common in patients with breathlessness, and cross-sectional population studies have shown considerable overlap in the causes of breathlessness.^11^ One study showed that 66% of people reporting breathlessness had ≥2 contributing causes to their breathlessness, with respiratory disease and anxiety or depression being the most common combination.^10^ The Australian National Breathlessness Survey found that obesity accounted for a quarter of breathlessness symptoms in adults.^12^ Deconditioning is a likely important contributing factor that is currently less well quantified.^13^

Despite the impact breathlessness has on quality of life,^14^ people with the condition may delay seeking help, normalising their symptoms,^15^^,^^16^ and frequently experience significant delays in diagnosis and treatment after seeking help.^17^^,^^18^

Evidence about patients’ experiences while awaiting diagnosis and clinicians’ experiences of managing the diagnostic challenges of chronic breathlessness in primary care is lacking. Previous literature has described the experiences of patients living with breathlessness in specific disease conditions, commonly cancer or cardiorespiratory disease.^15^^,^^19^^–^^22^ Patients have reported the ‘invisibility’ of breathlessness, which is difficult to describe and associated with stigma, leading to embarrassment about its effects.^23^ Patient help-seeking behaviour and clinician responsiveness to patients and their breathlessness have been described as important factors in being able to live well with breathlessness.^22^

**Table 1:** How this fits in

Delays to diagnosis for patients presenting with chronic breathlessness are well described. This study set out to investigate current care for patients awaiting a diagnosis to inform future diagnostic pathways. The data highlight the challenges of symptom recognition, timely investigations, making a positive diagnosis, and difficult consultations. To achieve earlier diagnosis and better outcomes for patients with breathlessness, clinicians need to Ask, Act, and Advise: Ask to understand and validate symptoms, Act to initiate timely investigations, and Advise a positive diagnosis while offering breathlessness relief strategies.

Challenges for clinicians around achieving a diagnosis include the complex multimorbidity of breathlessness,^16^^,^^24^ accessibility of investigations,^25^ and variable adherence to disease-specific diagnostic pathways.^18^ Surveys of respiratory clinicians indicate a greater focus on clinical features (such as cough and sputum) than on quality of life domains when assessing breathlessness.^26^ Interviews with respiratory medical trainees highlighted their concerns about discussing breathlessness with patients and symptom management.^27^ Overall, there is limited literature to provide insight into the reasons for delays to diagnosis for chronic breathlessness and possible solutions.

To develop diagnostic pathways to reduce delays to diagnosis and treatment, and to improve outcomes for patients, this study aimed to explore current usual care from the experiences of patients living with breathlessness seeking health care, and from primary care clinicians involved in the diagnostic process.

## METHOD

This research is reported in line with the standards for reporting qualitative research.^28^

### Participants

Semi-structured interviews were conducted between November 2019 and October 2021 with participants enrolled in a mixed-method feasibility cluster randomised controlled trial (cRCT) in England called Breathlessness DiagnosE Early in Primary care (Breathe DEEP) (ISRCTN14483247).^29^ The Breathe DEEP trial involved 10 GP practices across Leicester and Leicestershire, which were cluster randomised to either a structured diagnostic pathway (intervention) or to usual care. The structured diagnostic pathway included a panel of investigations, completed within 2 months of consultation with the GP for breathlessness. Usual care involved the clinician’s normal practice with national guidance highlighted.^30^ Eligible patients were >40 years old, breathless for longer than 2 months, and presenting for the first time, with no previous diagnoses for their breathlessness.^29^ Semi-structured interviews were conducted with clinicians from 10 practices enrolled in the cRCT, who were not necessarily directly involved in the care of the patients interviewed.

### Interviews

The patient interview guide (see Supplementary Box S1 for details) included questions on the patients’ experiences of help seeking for breathlessness and related health care, and the conduct of the cRCT. The clinician interview guide (see Supplementary Box S2 for details) explored diagnosis and breathlessness management, associated challenges, impact of the COVID-19 pandemic during this timeframe, and the conduct of the cRCT. Interview data relating to patient experience during lockdown at the start of the pandemic are published elsewhere.^31^ Data on the conduct of the cRCT are also reported elsewhere (article submitted for publication).

Interview guides were developed collaboratively with members of the local patient and public involvement (PPI) group. The PPI group helped with phrasing questions about how/when patients sought help for their breathlessness and their expectations. An iterative approach was taken throughout the interview and analysis process, including reviewing and amending the guide to explore issues arising in earlier interviews. Interviews with patients continued until perceived theoretical saturation; the topic guide was no longer evolving and data were of sufficient depth and complexity around the topic.^32^^,^^33^ Interviews with clinicians were completed until representation from each participating GP practice was achieved to maximise variation in responses.

Two interviewers, who were trained in qualitative methods, conducted interviews face to face or via telephone. Interviews were recorded and transcribed verbatim. One interviewer is a physiotherapist and the other has a non-clinical psychology background. Another researcher independently verified consistency of interview practice, communication style, and quality of data obtained by the interviewers.

### Analysis

The transcripts were analysed using reflexive thematic analysis,^34^ with analysis grounded in the interview data, supported by NVivo software (version 12). Analysis included familiarisation with data, generating initial codes, generating initial themes, reviewing and developing themes, refining and naming the themes, and producing the report.^34^ Initial coding was carried out independently by two researchers to develop a coding tree. One researcher completed full coding of all transcripts, maintaining a reflexive diary and discussions with the study team about the influences of previous experiences as a clinician working with patients with breathlessness. The wider research team discussed and reviewed the codes and patterns of shared meaning across the transcripts, drawing on experience and insight of the whole team, which included clinicians and researchers. The team collaboratively identified themes, using quotes from the transcripts to check data interpretation. The data were presented to the PPI group to check whether interpretation and description of the themes remained in line with the original data. One interviewer had not previously performed semi-structured interviews but was experienced in clinical consultations with patients presenting with breathlessness. This was balanced with a second interviewer who was not a clinician and had significant previous experience in qualitative research methods.

## RESULTS

Interviews were conducted with 34 patients and 10 clinicians. All patients consented to an interview as part of the wider Breathe DEEP study. Clinicians from each participating GP practice were asked to express interest in an interview, with the aim of gaining representation from each practice. Patients’ demographic details were as follows: 20 (59%) were female; mean age was 68 years (standard deviation [SD] 10.8, range 48–89); 32 (94%) were White British; and median indices of multiple deprivation quintile was 3 (interquartile range 2–5). Clinicians had a mean 17 years of experience (SD 6.3, range 6–30), five of whom (50%) were female, and seven (70%) were White British. Clinicians were from 10 GP practices with a mean population of 15 300 (SD 7.6, range 10–40); nine were GPs and one was a respiratory nurse. Five themes emerged from the data: recognising and validating symptoms of breathlessness is an important first step; clinical decision making for breathlessness is complex; difficult conversations arise when a disease-related diagnosis is not confirmed; disease management rather than symptom management is prioritised by clinicians; and patient experience is influenced by clinician communication style. The first four themes describe the patient and clinician journey, while the fifth theme about communication was evident throughout the first four themes.

### Recognising and validating symptoms of breathlessness is an important first step

Patients delayed presenting with breathlessness, often normalising symptoms. Patients described either the impact of exertional breathlessness on daily activities prompting them to seek help, or situations where they had sought help for another problem when their breathlessness was identified:

*‘I was getting out of breath, didn’t really do anything about it until people started commenting at work about it.’*

(Patient 30)

*‘But then I found that I was getting breathless if I just walked upstairs at home or if I walked from the lounge to the kitchen, and that’s when I thought I better go and see my GP.’*

(Patient 26)

*‘I went to see my GP about another problem … and then you say a throwaway thing, you know … “and I have problems with breathlessness”.’*

(Patient 33)

*‘It’s because I was getting these symptoms which I didn’t understand and I wanted somebody to go through it with me and just confirm whether I was ill or not.’*

(Patient 1)

Once patients sought help, they described being referred for investigation and how this helped to validate their symptom:

*‘I did find it really good because I was just thinking … “Well I’m only breathless” and I wasn’t really thinking it was anything bad, and then all of a sudden I was going for all these tests.’*

(Patient 7)

*‘I’d sooner have it tested and know than not have it tested and don’t know.’*

(Patient 16)

At the point of presentation to the GP, patient and clinician priorities were closely aligned in finding a cause for the breathlessness:

*‘I would like an answer … I think if you have an answer, you know where you’re going.’*

(Patient 7)

*‘Again because breathlessness is just a symptom, it’s not a diagnosis, so you need to look for a cause.’*

(Clinician 7)

Clinicians interpreted the validity of breathlessness in light of a diagnostic plan to identify a disease label:

*‘When a patient has genuine pathology, the barriers are not so difficult. So if you’ve got somebody who has got genuine COPD* [chronic obstructive pulmonary disease]*, genuine ischaemic heart beat, heart failure, or someone’s got asthma, we can test for that.’*

(Clinician 3)

*‘You may have a suspicion, for example, that it could be perhaps just down to some physical deconditioning but several differentials need to be excluded in order to confidently arrive at that diagnosis.’*

(Clinician 6)

### Clinical decision making for breathlessness is complex

Clinicians described balancing the need to investigate appropriately with avoiding unnecessary investigations because of concerns over patient burden and increased costs:

*‘In general practice we tend to try and be fairly focused. So you would try and do the investigations that you think are likely to show you what’s going on.’*

(Clinician 8)

*‘I think with every investigation you need to have a specific question in mind. You don’t want to investigate everyone because then that will have a massive strain … on the whole of the NHS.’*

(Clinician 4)

Clinicians described taking an incremental approach to diagnosis, as per their medical training:

*‘You are taught to say “Well, what’s the most likely diagnosis?”, so investigate for that and then revise your hypothesis in light of new information. So as a GP you’re taught that OK, so clinically I think this is COPD … Let’s arrange some spirometry and if that’s normal we’ll then revisit our hypothesis and revise our differential diagnosis.’*

(Clinician 10)

Clinicians described feeling less confident with a diagnosis of exclusion, when investigations returned with no abnormality, possibly finding it a trigger for onward referral:

*‘Because even if those investigations were normal, if you are looking at something like dysfunctional breathing, I can’t access any help for that in general practice … And I also I kind of want that reassurance from the consultant that I’m not missing anything as well.’*

(Clinician 10)

*‘Sometimes having been to a specialist clinic does reassure them more than I can, because they feel they’ve seen the expert in lungs, rather than a generalist GP.’*

(Clinician 8)

Patients expected to be sent for investigation or be told the cause of their breathlessness. Patients described different levels of investigation:

*‘And it feels like I’ve not had any answers anywhere really, well answers to rule out. Now I’d have liked more done to check the heart, especially as there’s three things there that is wrong.’*

(Patient 5)

*‘I think the best part of it, once you got the tests done, is getting a clear explanation of what is likely to be the problem, and equally what isn’t the problem.’*

(Patient 40)

### Difficult conversations arise when a disease-related diagnosis is not confirmed

All clinicians referred to difficult conversations with patients, often centred around causes of breathlessness without a disease label or where lifestyle advice and/or psychological support was required:

*‘It’s a challenge where you’ve got somebody who is symptomatic with breathlessness and you’ve investigated them for everything that you can think of and there is nothing positive coming back on their tests. And you believe that it’s probably due to deconditioning, weight gain, so where there’s not a specific pathology and patients are challenging you on that.’*

(Clinician 5)

*‘I think one thing I struggle with would be, if someone has got chronic breathlessness, diagnosing it due to their body habitus, or due to anxiety.’*

(Clinician 2)

*‘If you tell that it might be because of their weight, they know that they’re going to be told off. So I think they probably don’t want to believe that is the reason for the breathlessness.’*

(Clinician 1)

Some clinicians indicated they would engage in conversations that they recognised might be hard for patients to hear:

*‘For some patients I would diagnose the fact that they’re just unfit. They say they’re feeling breathless and you go “Well you’re fat and you do no exercise.” I wouldn’t say it quite like that, but there’s a reality check of the fact that you’re totally deconditioned.’*

(Clinician 7)

Patients also described difficult discussions, either in relation to the reason offered for their medically unexplained breathlessness, or when no explanation was forthcoming. One patient also reported not knowing he had a respiratory diagnosis:

*‘Told me that it was probably I wasn’t fit, they’ve done all the tests that they could, there was nothing wrong with me.’*

(Patient 28)

*‘And then when I went to see the nurse for my second breathing test she said, “How long have you had COPD?” I said, “What is COPD?” And he’d not told me that that’s what I’ve got. So I was in the dark, you know, I didn’t know I had anything wrong with me at all apart from infection.’*

(Patient 1)

*‘Everything gets put down to menopause or anxiety, I have anxiety problems, I did have menopause, I’m past that.’*

(Patient 40)

Clinicians and patients also referred to time pressures in a consultation, both in relation to discussions with patients and to investigating in a timely manner:

*‘The disadvantage is we are time pressured and you generally have ten minutes for an appointment. Which isn’t a long time to do a thorough examination, history, explanations, and all the things that go with it.’*

(Clinician 8)

*‘And I guess in that time the patient and the doctor can get a bit frustrated, well we’re not finding anything, we’ve not found the cause of this yet, if you’re not getting your tests done in a timely way.’*

(Clinician 10)

*‘Don’t think it was thorough, well they can’t be thorough can they because at the end of the day they’ve got next one in?’*

(Patient 6)

### Disease management rather than symptom management is prioritised by clinicians

Although the need to find a cause for breathlessness was a clear and appropriate priority for both clinicians and patients, the data also highlighted a lack of advice for immediate symptom management during the diagnostic journey:

*‘They’ve not given me any treatment to help it because it’s not diagnosed, which is what I found a bit odd. I mean they could have given me something to just tide me through a bit.’*

(Patient 5)

*‘Just help to know, to be a bit more aware of what I can do to live with this.’*

(Patient 31)

Some clinicians acknowledged the importance of symptom management:

*‘Because our primary concern would be the patient’s wellbeing and trying to make the patient feel better and give them some symptomatic relief.’*

(Clinician 4)

*‘Advice would be around lifestyle, so BMI* [body mass index]*, smoking, and just trying to exercise regularly would be a general advice; but also safety netting advice as well.’*

(Clinician 6)

*‘I suspect that I would have a trial of treatment and see if that made a difference, think about trying an inhaler with their symptoms.’*

(Clinician 7)

However, many clinicians expressed lower confidence in suggesting non-pharmacological approaches to help with breathlessness:

*‘In terms of breathing exercises and mindfulness, and that side of things, I think I’m probably not in a particularly good position to advise patients very well.’*

(Clinician 3)

*‘For me personally, I don’t know that I necessarily give breathlessness management advice in terms of breathing techniques and things like that. I don’t know that I give that initially. It probably ends up happening once you get the actual formal diagnosis of COPD and then we maybe get into the disease management side of things.’*

(Clinician 10)

### Patient experience is influenced by clinician communication style

Patients described a variety of clinician communication styles, and this theme ran throughout the patient journey. Several patients described the benefits of clear explanations and where they had positive experiences:

*‘To be honest with you, it’s all been very good. Very clear, concise of just exactly what’s happening. This is what we’re going to do. I quite like doctors being very straightforward.’*

(Patient 50)

*‘The follow-up side of it, they were very good … especially the doctor, she didn’t hang about … it all fell into place.’*

(Patient 29)

*‘And I think it was taking that time to say “OK yes … these are other things that need to be checked, these are tests that should have been done, so I’m going to get them done”, and just taking that time to listen.’*

(Patient 40)

However, there were examples of patients feeling dismissed or embarrassed. These contrasting styles of delivering information and responding to patients’ concerns also occurred at the point of diagnosis:

*‘*[GP] *said, “Well you are overweight”, just like that and that was it.’*

(Patient 47)

*‘*[GP] *said, “I think you’re just getting anxious” and … that I need to just relax. And they* [GP] *said, “When the heart scan’s done we’ll then discuss it.”’*

(Patient 44)

*‘I feel my next breath is going to be my last one. And his comments were “Well that’s a bit extreme” … he was very professional, but it was very short.’*

(Patient 24)

*‘“Nothing the matter with you, it’s just because you’re not fit” … I feel a fraud going, you know, I just don’t want to go and be spoke to like that.’*

(Patient 28)

Clinicians revealed their challenges in responding to patients’ concerns and they described ways to discuss breathlessness with patients, particularly where a diagnosis is not straightforward:

*‘By explaining why you’ve taken it seriously, you’ve excluded other causes, you’re allowing them to come on that journey with you … And usually they come along with you, I think partly because they feel listened to.’*

(Clinician 8)

*‘“But I’ve got COPD” and you say “Yeah but unfortunately you are quite overweight and … with obese patients you will get short of breath and especially if your levels of fitness are not very good.” And it’s hard to engage them to take some sort of responsibility for improving their overall health, they just want to be given an inhaler that’s going to sort everything out.’*

(Clinician 9)

## DISCUSSION

### Summary

This study examined the experiences of current care in 2020–2021 for patients and primary care clinicians navigating the journey from presentation with breathlessness to a possible diagnosis. The data provide novel insights into possible reasons for the delays to diagnosis for patients presenting with breathlessness. The combination of poor symptom recognition and delayed help seeking by patients, alongside an incremental approach to investigations, could lengthen the diagnostic process. Clinicians described challenges in explaining breathlessness associated with deconditioning when specific disease states had been investigated and excluded. This frequently led to difficulty in progressing to effective breathlessness management. Throughout the diagnostic journey, communication was key and there was clear indication that time pressures in primary care and lack of specialist support were an issue. While awaiting investigative results, provision of advice or information about low-risk, evidence-based strategies for relief of breathlessness appeared to be an unmet need for patients. To achieve better outcomes for patients with breathlessness, measures are needed to address the reasons for delays to diagnosis, to improve early accurate diagnosis, and to offer breathlessness relief strategies.

### Strengths and limitations

To the authors’ knowledge, this is the first study to report usual care through the experiences of patients with chronic breathlessness awaiting a diagnosis, alongside the experiences of clinicians managing the diagnostic process. Thirty-four patients were interviewed, providing a sufficient sample size for data analysis.

The ethnicity of most patients was White British, which is not representative of the diverse local population. Opportunistic recruitment at the point of consultation with the GP was used for the study and either adults from other ethnic backgrounds declined to have their details passed on to the study team or were not invited by their GP. Of the participants identified, two were excluded because they had insufficient understanding of English, therefore interpretation should be offered for future studies. The number of clinician interviews was limited as a result of pressures in primary care during the COVID-19 pandemic. One clinician from each of the GP practices in the study was interviewed, but their views may not be representative. The sampling for clinician interviews was mostly opportunistic, possibly introducing positive responder bias. It is not possible to exclude that being involved in the wider Breathe DEEP trial may have influenced patients’ and clinicians’ experiences.

### Comparison with existing literature

As in this study, others have found that breathlessness is likely to be identified by clinicians when patients are seeking help for a different problem, thus increasing the complexity of patient assessment in an already time-limited appointment.^35^ Some patients recalled being prompted by others to seek help, supporting existing literature around help-seeking behaviour by patients with breathlessness.^15^^,^^19^

The incremental approach to investigation to rule out individual diagnoses described by clinicians may be due to disease-specific guidelines that promote excluding a particular diagnosis, rather than a holistic approach to find all causes of a symptom.^36^^,^^37^ An incremental approach could be appropriate if timely investigations and multiple reviews were possible, but this is commonly not the case and has been further exacerbated by the COVID-19 pandemic causing delays in health care.^38^

There are multiple causes of, and contributing factors to, breathlessness, which may be missed when a single disease diagnostic algorithm is used. The current study highlights the tension between performing extensive investigations in every patient to find a diagnosis rapidly versus patient burden, and overuse and cost; some investigations may also have a level of harm involved.^39^^,^^40^

Clinicians described challenges when a disease label was not identified, feeling ill prepared to offer advice or be definitive about weight, deconditioning, and anxiety as causes of breathlessness. These causes were considered less acceptable as a positive or confirmed diagnosis to both clinicians and patients. However, consideration of the definition of a diagnosis: *‘the process of determining the nature of a disorder by considering the patient’s signs and symptoms, medical background, and — when necessary — results of laboratory tests’*^41^ highlights that the nature of the disorder can be a multitude of things and is not solely a disease label.

Several quotes in this study demonstrate that clinicians and patients recognised that deconditioning and obesity were important factors contributing to breathlessness. Opportunistic behaviour change interventions, such as smoking cessation, increased physical activity, and weight loss, are advocated in healthcare settings; however, a lack of time and perceived lack of clinician skills and patient motivation are known barriers.^42^ Time was a factor identified by participants in this study. Successful supervised exercise programmes, such as pulmonary and cardiac rehabilitation, are designed for adults with respiratory or cardiac conditions with exertional breathlessness;^36^^,^^43^ deconditioning is frequently the dominant cause of breathlessness, rather than the specific pulmonary or cardiac impairment, and the mechanism of benefit of rehabilitation is through the effect on skeletal muscle. Research is ongoing to broaden these programmes for adults with exertional breathlessness regardless of underlying disease.^44^ Recognising and describing deconditioning as a positive diagnosis with a targeted therapy, such as exercise training, may aid communication between clinician and patient.

### Implications for practice

This study found that there are challenges associated with reaching a meaningful diagnosis for patients with breathlessness, but that recognition and validation of the symptom of breathlessness is an important first step. Time is a constant pressure in GP consultations; however, time spent early on listening to patients’ concerns about how breathlessness affects them may improve the overall patient experience.^45^^,^^46^

The clinician’s communication style has a profound effect on the patient, and training around advanced communication skills, opportunistic behaviour change, and motivational interviewing have been shown to have some benefit.^47^^,^^48^ Providing patients with information about the investigation process and about what has been excluded may be of therapeutic benefit in reducing their anxiety about serious underlying pathology. However, this needs to be balanced with receipt of a positive diagnosis that the patient understands.

It is important for clinicians to consider breathlessness as a therapeutic target, as well as an important indicator of underlying illness.^49^ There are models to support this approach, which facilitate clinicians in enabling patients to develop coping strategies and manage their symptoms.^16^^,^^22^ A symptom-based approach is commonly used with chronic pain,^50^ which like breathlessness is a complex symptom with affective and sensory components.^51^ Previous comparisons of clinicians’ approaches to chronic pain and breathlessness suggest that breathlessness was less likely to be objectively assessed, followed up, and perceived as requiring additional treatment than chronic pain.^52^ Healthcare approaches to chronic pain serve as a good example of a symptom-based approach to holistic assessment, diagnosis, and management.

This study indicates that clinicians value specialist support in instances where a diagnosis is less straightforward. Understanding the most effective way to communicate the key messages to patients when the diagnosis for breathlessness is deconditioning, obesity, or anxiety, or where other conditions have been excluded, alongside a personalised symptom-based management plan, could also be of benefit.

A complex intervention is needed to support patient and clinician recognition of breathlessness, early symptom-based investigation that allows for the detection of multiple causes, and support for clinicians to be confident with delivering less disease-specific diagnostic labels such as deconditioning.

## Data

The datasets generated and analysed during the current study are available from the corresponding author on reasonable request.
